# Dimensional transformation of chemical bonding during crystallization in a layered chalcogenide material

**DOI:** 10.1038/s41598-020-80301-5

**Published:** 2021-03-08

**Authors:** Yuta Saito, Shogo Hatayama, Yi Shuang, Paul Fons, Alexander V. Kolobov, Yuji Sutou

**Affiliations:** 1 Device Technology Research Institute, National Institute of Advanced Industrial Science and Technology, Tsukuba Central 5, Higashi 1-1-1, Tsukuba, 305-8565 Japan; 2 Department of Materials Science, Graduate School of Engineering, Tohoku University, 6-6-11 Aoba-yama, Sendai, 980-8579 Japan; 3 Department of Electronics and Electrical Engineering, Faculty of Science and Technology, Keio University, 3-14-1 Hiyoshi, Kohoku-ku, Yokohama, Kanagawa 223-8522 Japan; 4 Department of Physical Electronics, Faculty of Physics, Herzen State Pedagogical University of Russia, 48 Moika Embankment, St Petersburg, 191186 Russia

**Keywords:** Electronic materials, Two-dimensional materials

## Abstract

Two-dimensional (2D) van der Waals (vdW) materials possess a crystal structure in which a covalently-bonded few atomic-layer motif forms a single unit with individual motifs being weakly bound to each other by vdW forces. Cr_2_Ge_2_Te_6_ is known as a 2D vdW ferromagnetic insulator as well as a potential phase change material for non-volatile memory applications. Here, we provide evidence for a dimensional transformation in the chemical bonding from a randomly bonded three-dimensional (3D) disordered amorphous phase to a 2D bonded vdW crystalline phase. A counterintuitive metastable “quasi-layered” state during crystallization that exhibits both “long-range order and short-range disorder” with respect to atomic alignment clearly distinguishes the system from conventional materials. This unusual behavior is thought to originate from the 2D nature of the crystalline phase. These observations provide insight into the crystallization mechanism of layered materials in general, and consequently, will be useful for the realization of 2D vdW material-based functional nanoelectronic device applications.

## Introduction

Two-dimensional (2D) van der Waals (vdW) materials are characterized by covalently bonded Angstrom-length scale atomic layers located within the 2D x–y planes of the crystal structure^1^. There is, of course, interatomic interaction along the z-axis in the form of vdW bonding also known as secondary bonds, but this form of bonding is much weaker in terms of bonding energy than primary bonds i.e. covalent bonds within layers. Consequently, there is inherent chemical bonding anisotropy in the crystal structure along the x, y, and z axes. Interlayer interactions along the z-axis are weak and such materials are typically stable even in monolayer form (Angstrom-scale) resulting in the epithet 2D materials. Representative 2D vdW materials include graphene^2^, transition metal dichalcogenides (TMDs)^3^, boron nitride^4^, black phosphorus^5^, and topological insulators such as Sb_2_Te_3_^6^. In addition to typical composition dependent electronic material properties such as insulating, semiconductor, semimetal, or metallic behaviors, the unusual electronic, optical, and magnetic properties of 2D vdW materials have generated interest in a wide range of research fields from fundamental to applied science^7–9^. The emerging functionality arising from the atomic-layer scale structure of these materials holds the promise of the ultimate nanotechnology for future devices.

Most research studies on 2D vdW materials have focused on the crystalline phase. Although disorder in 2D vdW materials such as point defects or grain boundaries has been explored, structural information on the disordered state of 2D vdW materials has yet to be understood^10^. In particular, the amorphous phase of 2D vdW materials has been overlooked. Generally, chemical bonding in the amorphous phase is random and isotropic. In other words, the chemical bonding dimensionality of the amorphous phase is 3D. Therefore, the fundamental question arises as to how the amorphous-to-crystal transition (or simply crystallization) occurs between two phases with different chemical bonding dimensionality. In this work, the structural analysis of Cr_2_Ge_2_Te_6_ films in a variety of forms ranging from amorphous to crystalline was undertaken.

Cr_2_Ge_2_Te_6_ has been reported to exhibit intrinsic ferromagnetism in its 2D crystalline form with a Curie temperature of about 65 K^11–13^. Additionally, reversible phase-change between the amorphous and crystalline phases has been demonstrated for non-volatile phase change memory applications^14–17^. Furthermore, pressure-induced amorphization of Cr_2_Ge_2_Te_6_ has been recently reported^18^. In particular, understanding the crystallization mechanism is crucially important for phase change memory applications as the amorphous-crystal transition is used more than 10^7^ cycles in typical devices^19^. Conventional phase change materials (PCMs) such as Ge–Sb–Te alloys undergo crystallization from the amorphous to the metastable cubic phase followed by a phase transition to the stable hexagonal phase^20^. One remarkable aspect of Cr_2_Ge_2_Te_6_ is that it contains the transition-metal, Cr, whereas typical PCMs form their covalent bonds using *p*-electron from the constituent Group IV (Ge), V (Sb), and VI (Te) elements. The important role of *d*-electrons in the phase change process as well as promising features of transition-metal based PCMs have been already pointed out^21–24^. Therefore, the crystallization mechanism of transition-metal based layered Cr_2_Ge_2_Te_6_ is expected to be different from conventional materials.

The theory of crystallization is quite old, dating back more than 80 years^25^, but the process of crystallization is still attracting attention in a variety of materials science fields such as one-dimensional long-range ordered crystals in some particular oxides^26^, or the observation of early-stage crystal nucleation in metal nanoparticles^27^. The fact that these papers were published this year underscores the rapid development of this field even now.

In this work, two different structural probes with differing coherence lengths were used to understand the structural evolution of a Cr_2_Ge_2_Te_6_ film from the 3D-bonded amorphous to the 2D-bonded crystalline phase. Extended X-ray absorption fine structure (EXAFS) spectroscopy is well known as a powerful tool for examining the local structure of materials. The technique is applicable, in principle, to any solid phase including amorphous or glassy phases and even liquid phases due to its nanometer scale coherence length. On the other hand, X-ray (Bragg) diffraction (XRD) is a useful analytical technique to identify the structure of a material over longer length scales due to its longer (microns or greater) coherence length leading to the technique often being used to determine the crystal structure of materials in which the atomic arrangement is required to have long-range order. Even though this technique is also essential for the structural analysis on disordered materials such as glasses and liquids, the absence of sharp peaks makes the data analysis significantly more complicated and the obtained information is often qualitative. For example, the use of X-ray diffraction provided the first direct proof of the structural origin of reversible photodarkening in chalcogenide glasses^28^, but stayed short of defining any details of the structural changes. In fact, the combination of XRD and EXAFS is a powerful approach to investigate structures with local distortions that would otherwise be obscured by the averaging effects of longer coherence length probes, as was previously demonstrated for GeTe^29^.

By carefully analyzing structural measurements of Cr_2_Ge_2_Te_6_ films annealed at different temperatures, an intermediate metastable “quasi-layered” state between the amorphous and crystalline phases that exhibits “long-range order co-existing with short-range disorder” in atomic alignment was identified. This is counterintuitive in that long-range order is typically a consequence of the long-range coherence of short-range order, suggesting that the atomic arrangement of a system with long-range order should also be ordered on a short-range length scale. We show that such unusual behavior can arise due to the inherent tendency of layered materials to form a quasi-layered atomic alignment during crystallization. These observations constitute a significant step forwards toward understanding the crystallization mechanism of transition-metal containing layered chalcogenide materials and the application of these materials to future nano-scale electronic device development.

## Results and discussion

**Figure 1:**
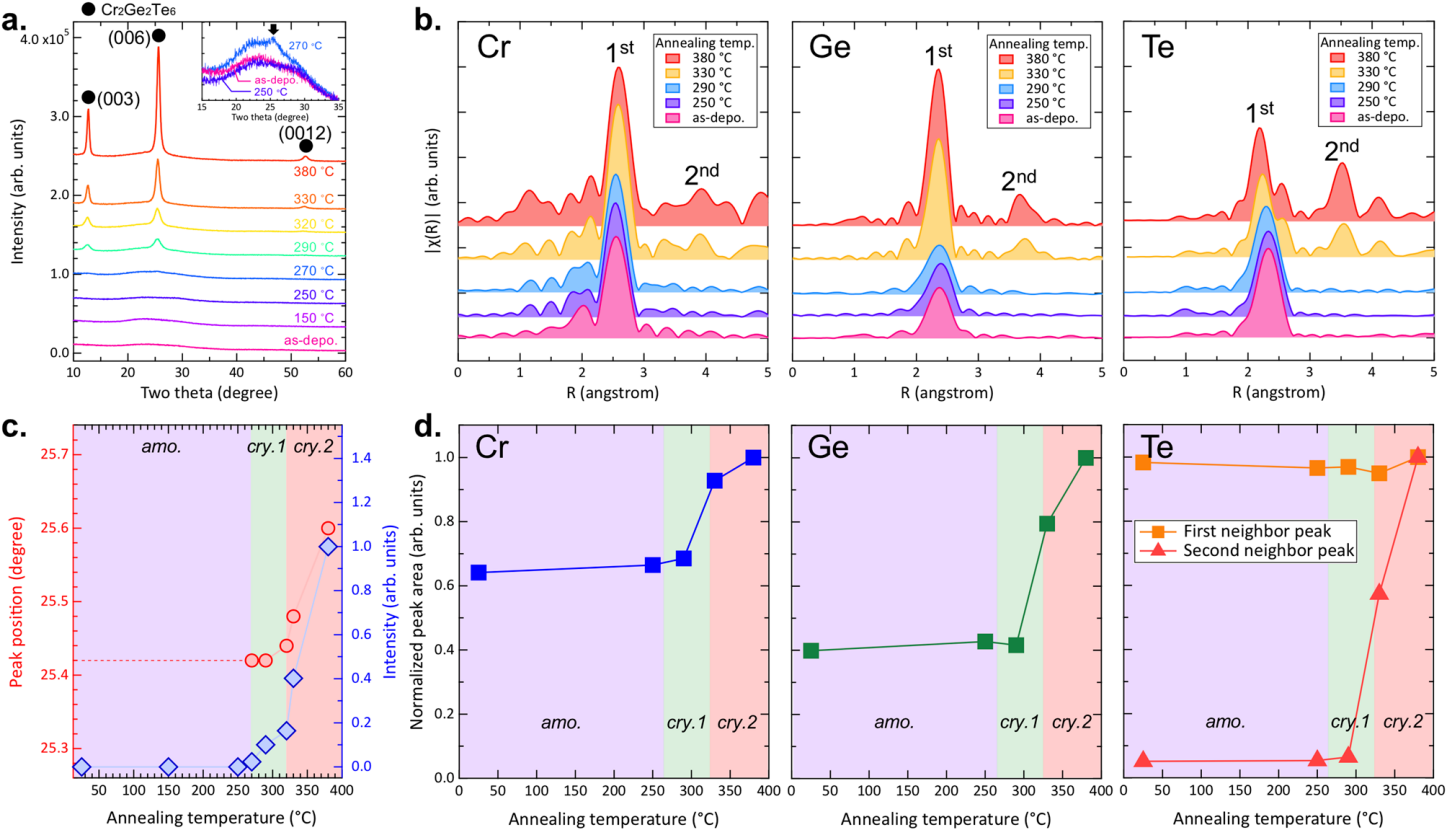
Experimental (**a**) XRD and (**b**) Fourier transformed EXAFS spectra of Cr_2_Ge_2_Te_6_ films for different annealing temperatures. Inset of (**a**) shows magnified XRD patterns for the as-deposited, 250 °C-, and 270 °C-annealed films. The onset of crystallization can be confirmed by the appearance of a new peak at 270 °C. Annealing temperature dependence of (**c**) the XRD (006) reflection position and intensity, and (**d**) the peak area of EXAFS spectra for each element.

XRD patterns of CrGT films taken as a function of annealing temperature are shown in Fig. 1a (some data have been taken from Ref.^14^). It can be clearly seen that the amorphous phase is characterized by a broad XRD reflection that remains unchanged for annealing temperatures up to 250 °C, whereupon additional reflections appear after annealing at 270 °C as can be seen in the inset of Fig. 1a indicated by the black arrow. All reflections can be assigned to the series of (*00l*) planes of the trigonal Cr_2_Ge_2_Te_6_ crystal structure suggesting that the crystallized films are highly oriented along the *c*-axis, in other words, the vdW gaps lie parallel to the substrate surface. This trend remains unchanged for annealing temperatures up to 380 °C. According to a previous report, in which differential scanning calorimetry (DSC) measurements were performed on Cr_2_Ge_2_Te_6_ films, an exothermic peak corresponding to crystallization was observed between 270 and 290 °C, an observation consistent with the current results^14^.

Fourier transformed Cr, Ge, and Te K-edge EXAFS spectra are shown in Fig. 1b. Raw data of EXAFS measurements are available in Supporting Information Fig. S1. EXAFS spectra were analyzed and information on the local structure, such as bond lengths and coordination numbers, were reported elsewhere^30^. Only the first nearest neighbor peak can be seen around 2.5 Å for all elements for annealing temperatures up to 290 °C, followed by the appearance of second nearest neighbor peaks between 3 and 4 Å for samples annealed at 330 °C or greater. Since the presence of a second nearest neighbor peak suggests a medium- or long-range ordered atomic arrangement, it does not appear in a highly disordered system, i.e. the amorphous phase. On the other hand, it is known that the first nearest neighbor peak is present even in disordered systems as in such systems ordering is not truly random and short-range order remains. Therefore, the appearance of a second nearest neighbor peak in the EXAFS spectra is usually interpreted as a sign of crystallization *i.e.* the formation of long-range order. It should be noted that the first nearest neighbor peak of Te splits into two at 330 °C, the same annealing temperature for which the second nearest neighbor peak appears suggesting that the changes in Te *K*-edge spectra are correlated, reflecting the evolution of the local structure upon annealing. The origin of the splitting of the Te first neighbor peak will be discussed later.

An important conclusion to be drawn from these two different structural measurements is that the temperatures, at which the profiles drastically change, are different, namely, the XRD spectra undergo changes between 270 and 290 °C, while the EXAFS spectra suggest that the most significant changes occur between 290 and 330 °C. These seemingly contradictory results imply that the phase change process of Cr_2_Ge_2_Te_6_ film is not as simple as a general amorphous-crystalline transition, but instead, that there may exist another “phase” in addition to amorphous and crystalline phases. Here, in this work, we define these phases as amorphous (*amo*.), crystalline 1 (*cry.1*), and crystalline 2 (*cry.2*).

Figure 1c shows the annealing temperature dependence of the two-theta position as well as the intensity of the XRD (006) reflection taken from (a). The three different phases defined above are shown in different colors. According to the XRD results, the reflections appear for annealing temperatures greater than 270 °C, but the peak position and intensity do not change drastically over the temperature range of *cry.1*. However, once the sample has been annealed above 330 °C (*cry.2*), a significant peak shift to higher angles as well as enhancement in intensity are observed. It should be noted that the ideal, stress-free peak position of (006) reflection is 25.968°^31^. Therefore, the observed peak shift to higher angle starting from about 25.4° corresponds to a decrease in the lattice spacing as the system approaches the ideal, stress-free value due to internal stress relaxation during the annealing process. The enhanced reflection intensity is thus a consequence of improved crystallinity due to high temperature annealing.

The normalized peak areas for the EXAFS spectra have been plotted in (d) for each temperature range. For Te, the area of the second nearest neighbor peak is also shown. It was found that the peak areas were almost identical for the *amo.* and *cry.1* phases and only significantly change when annealed above 300 °C (*cry.2*). As summarized in Fig. 1, the two different techniques suggest two different crystallization onsets, namely, the XRD results indicate crystallization takes place around 270 °C (between the *amo.* and *cry.1* phases), while the second nearest neighbor peaks are present only for annealing temperatures greater than 300 °C in EXAFS (between *cry.1* and *cry.2*).

**Figure 2:**
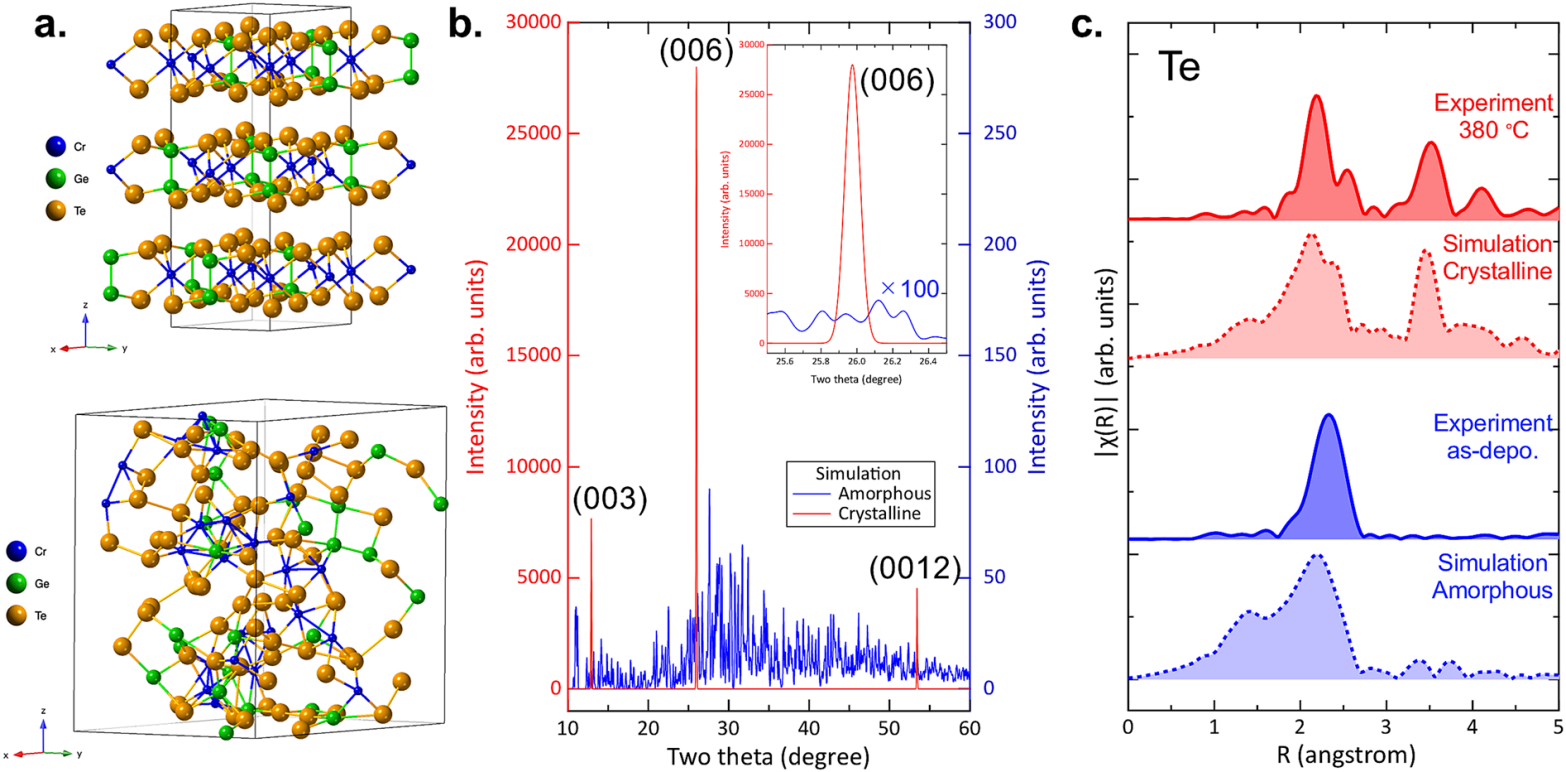
(**a**) (Top) Ideal crystal structure and (Bottom) simulated amorphous model. (**b**) Simulated XRD patterns of both phases. Inset magnifies the two theta range corresponding to the 006 reflection. (**c**) Simulated EXAFS spectra for comparison with the experimental results.

Before delving into the detailed phase change mechanism discussion, we compare the experimental XRD and EXAFS results with simulated results for the amorphous and crystalline phases. The upper panel of Fig. 2a shows the ideal crystalline structure of Cr_2_Ge_2_Te_6_, while the bottom panel displays a melt-quenched model obtained from DFT. The crystalline Cr_2_Ge_2_Te_6_ phase is a layered structure with vdW gaps in which Te atoms terminate each vdW block. This layered 2D feature is completely absent in the melt-quenched amorphous model, which forms in a 3D-bonded random atomic arrangement.

Simulated XRD patterns of these two models are shown in Fig. 2b. For the simulation of the crystalline phase (Fig. 2a), a (*00l*) preferred orientation was assumed as the experimental XRD results demonstrate that the crystallized film exhibits a strong *c*-axis preferred orientation. It can be seen from Fig. 2b that the crystalline model shows strong and sharp reflections, while reflections have negligible intensity in the amorphous model. The inset image magnifies the two-theta range in the vicinity of the (006) reflection showing the complete absence of such a reflection in the amorphous model. The absence of the (006) reflection is a consequence of the lack of long-range atomic order in the disordered system. These results are consistent with the experiment results shown in Fig. 1a.

Fourier transformed Te *K*-edge EXAFS spectra were calculated and compared with experiment as can be seen in Fig. 2c. In the crystalline phase, the splitting of the first reflection as well as the presence of a second reflection were well-reproduced by the simulations. Moreover, the single peak feature of the first peak and the absence of a second peak are also in good agreement for the amorphous phase. It should be noted that the shoulder peaks appearing between 1 and 1.5 Å in the simulations are a consequence of the tails of the XAFS scattering function and do not imply the presence of chemical bonding in this distance range. Figure 2 suggest that the simulation results for the both the amorphous and crystalline models reproduce well the experimental XRD and EXAFS data. The question then arises as to the structure of the experimental intermediate phase *cry.1* that exists in the annealing temperature range between the two extremes.

**Figure 3:**
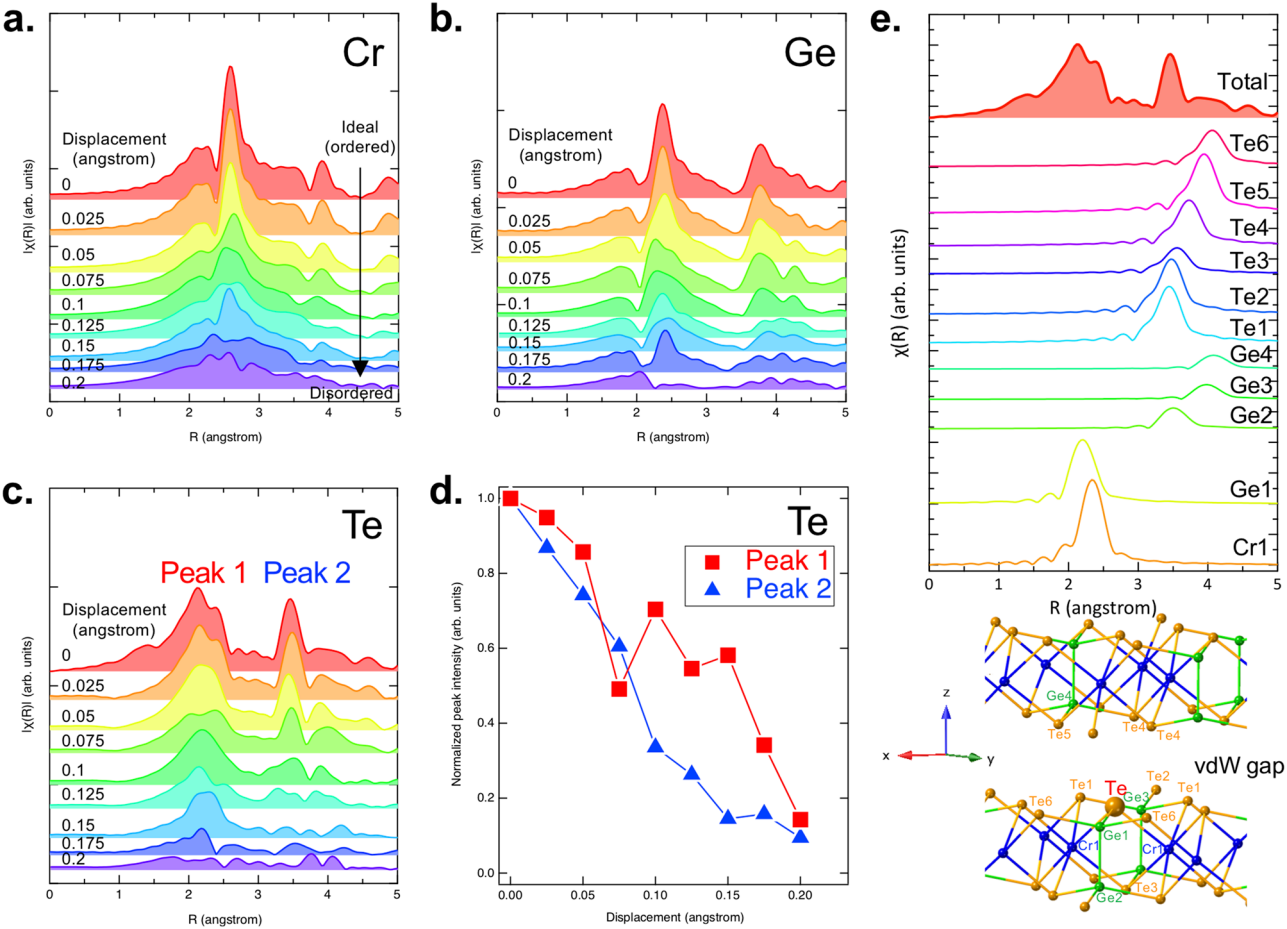
Simulated EXAFS spectra as a function of the displacement of atoms from their ideal position for (**a**) Cr, (**b**) Ge, and (**c**) Te. (**d**) Displacement dependence of the peak area for Te. Note that atoms were displaced randomly within the x–y plane. (**e**) (Top) Deconvoluted Te spectrum for the ideal crystal model. (Bottom) A crystal structure model with labels for each atom.

To determine the details of the intermediate phase that exists between the ideal crystal and melt-quenched amorphous models, the fractional coordinates of each atom were randomly displaced from their original positions in the ideal structure using the python library pymatgen^32^. Several possible scenarios for the atomic displacement were investigated and it was concluded that the structural model that best-reproduced experiment was that for which all elements were displaced randomly within the x–y plane under the constraint that the z-axis coordinate remained fixed. The evolution of the simulated EXAFS spectra for each element is shown in Fig. 3a–c. The results from other disordering scenarios can be seen in Supporting Information Fig. S2. Starting from the ideal crystal structure, all atoms were randomly displaced within the x–y plane over the range of 0.025 to 0.2 Å from the original positions and EXAFS spectra were simulated for each given displacement value. The most important experimental feature observed at 290 °C is that only the first peak is present, the second peak being absent. Figure 3d shows the simulated Te peak intensities for Peaks 1 and 2 as a function of atomic displacement. It can be clearly seen that over the displacement range from 0.1 and 0.2 Å, Peak 1 remains relatively strong while a sharp decrease in Peak 2 intensity is observed. The distinctive difference in peak intensities matches the experimental results well. Furthermore, the splitting of Peak 1 into two peaks at the Te edge (Fig. 3c) was found to vanish with increasing displacement, suggesting the same trend as can be seen in Fig. 1b. On the other hand, the intensities of Peaks 1 and 2 also drastically decreased in a similar manner when different scenarios were considered, for instance, displacing all atoms randomly in x, y, and z directions (Supporting Information Fig. S2). It should be noted that the simulations were carried out using a rather simple paradigm while the actual situation may be more complicated, but it can be deduced from the simulations that atomic displacement within the x–y planes is dominant during the phase change process.

The contribution of each scattering path to the overall EXAFS spectra is now discussed in detail. Here, a (backscattering) scattering path consists of a combination of two atoms, namely, the absorption center and the neighboring atom from which the outgoing photoelectron wavefunction is scattered. In Fig. 3e bottom, as an example, the absorption center Te, has been enlarged, and the neighboring scatters have been labelled. The top panel of Fig. 3e shows the individual single-scattering path contributions to the simulated EXAFS spectra as can be seen in the bottom panel. It can be clearly seen that Te–Cr1 and Te–Ge1 paths dominate the first nearest neighbor peak. Since the bond lengths of Te–Cr and Te–Ge slightly differ, the Te–Cr1 and Te–Ge1 paths reach maximums at different positions in **R** resulting in the apparent peak splitting of the first nearest neighbor peak. The second peak was found to mainly consist of Te–Te bonding with individual path contributions labelled as Te1–6. As can be seen at the bottom of Fig. 3e, Te–Te bonds are formed within the same vdW layer as well as in the vdW gaps. This suggests that once Te atoms are displaced from their ideal positions, the coherence originating from short (or medium)-range order rapidly attenuates resulting in the absence of a second nearest neighbor peak. Here, it should be emphasized that the degree of freedom in the displacement direction cannot be totally random as random atomic movement in any direction would otherwise result in the system being in the amorphous phase, which would in turn not give rise to XRD reflections as mentioned in the discussion of Fig. 2. Instead, as demonstrated by the simulated EXAFS spectra shown in Fig. 3a–c, disorder being predominantly confined within the x–y plane leads to a more reasonable reproduction of the experimental results.

**Figure 4:**
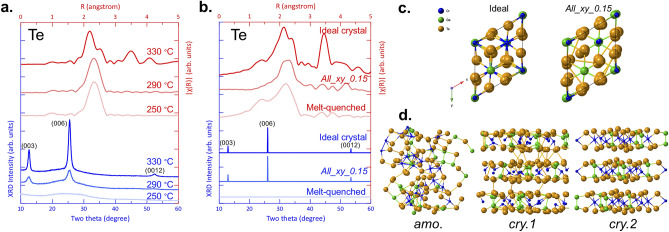
(**a**) Experimental XRD patterns and EXAFS spectra for different annealing temperatures. (**b**) Simulated XRD and EXAFS results for models with different atomic arrangements. (**c**) Top-view for the ideal and *All_xy_0.15* models from the z-axis. (**d**) Schematic evolution of crystallization for Cr_2_Ge_2_Te_6_ with three representative stages.

Finally, based upon the obtained results, we propose a possible crystallization mechanism for amorphous Cr_2_Ge_2_Te_6_ with respect to the chemical bonding dimensionality change. Figure 4a shows two experimental results, XRD and EXAFS, for films annealed at three different temperatures. From the XRD point of view (Fig. 4a blue), the 290 and 330 °C samples can be categorized into the same group, namely, a crystalline state characterized by the appearance of well-defined XRD reflections. On the other hand, the broad profile of the 250 °C sample exhibits the typical characteristics of an amorphous phase. A similar trend can be seen in the EXAFS spectra (Fig. 4a red) in that the 250 and 330 °C samples are amorphous and crystalline phases, respectively, which are characterized by the absence and presence of a second nearest neighbor peak, respectively. The EXAFS spectrum of the 290 °C sample, however, indicates an amorphous phase as it lacks a second nearest neighbor peak. This is counterintuitive as the XRD pattern for the same sample exhibits a clear signature of the crystalline phase. Here, we would like to remind the reader of the classification of a solid with respect to its atomic arrangement. A crystal exhibits both short- and long-range order in its atomic arrangement which is typically described as being a consequence of translational symmetry. On the other hand, disordered materials such as amorphous solids or glasses have no long-range order as the name “disordered” implies, while still typically retaining short-range order similar to that of the crystalline state. XRD (Bragg) diffraction measurements rely on the long-range coherence of the atomic arrangement making the technique useful to analyze crystal structures and to distinguish between crystalline and amorphous phases. EXAFS is a powerful tool for the analysis of the local structure of a material as its coherence length is much shorter than XRD, typically on the order of a nanometer. Summarizing the experimental results, the 290 °C sample exhibits long-range order (according to XRD) in the absence of short-range order (according to EXAFS), a very unusual pattern.

The simulations well-reproduced this situation as shown in Fig. 4b. Here, three structural models were considered. The first was the ideal crystalline structure, while the second was a melt-quenched model from ab initio molecular dynamics simulations representing the amorphous phase. The intermediate model was obtained by displacing all atoms 0.15 Å from the ideal crystalline structure in a random direction within the x–y plane as described in Fig. 3, hereafter, this model is referred to as *All_xy_0.15*. The ideal crystal model clearly shows a second nearest neighbor peak as well as XRD reflections, while only the first nearest neighbor peak in EXAFS is present and no reflections appear at all in the simulated XRD for the melt-quenched model representing the amorphous phase. Both simulated results are in good agreement with the experimental results for the 330 °C (crystal) and 250 °C (amorphous) annealed films. Intriguingly, the *All_xy_0.15* model also lacks a second nearest neighboring peak similar to the melt-quenched model, while at the same time exhibits clear reflections in the XRD simulation that resemble those of the crystalline model being consistent with experimental results (Fig. 4a). These results can be understood by recalling that XRD is insensitive to local fluctuations in the atomic arrangement.

The *All_xy_0.15* model along the z-axis is compared with the ideal model in Fig. 4c. It can be seen that all atoms slightly deviate from their ideal positions, and this significantly affects the EXAFS spectra as the coherence of the backscattered wave functions is destroyed due to the variation in scattering path lengths to second nearest neighbor atoms. Note that even though the x and y coordinates were displaced, the z values remained fixed at their original positions in the simulation, resulting in the appearance of XRD reflections as strong as those of the ideal model in (b). The intensity of XRD reflections from (*00l*) planes is not affected by displacement in the x and y coordinates as only the z spacing is crucial in determining the XRD intensity of the (*00l*) reflections as the film is strongly-oriented. The real situation may be more complicated in that the z coordinates likely deviate from the ideal values as well. This is confirmed by the weaker XRD reflection intensity shown by the 290 °C annealed film versus the 330 °C annealed film as can be seen in Fig. 4a suggesting that the z coordinate does not assume the ideal values of the crystalline phase during the crystallization process. It should be noted that even in the ideal structure the Te sublattice is strongly disordered within the x–y plane, while Cr and Ge atoms are ordered in all layers. This suggests that the position of Te atoms depends on its neighbors reflecting the difference between Cr–Te and Ge–Te bond lengths. Therefore, even when a layer-like structure is formed during the annealing process, the chemical disorder of cation atoms within a single layer also affects the intensity of the second nearest neighbor peak.

A schematic outline of the crystallization process of Cr_2_Ge_2_Te_6_ is shown in Fig. 4d. The as-deposited film is in an amorphous state (*amo*.) with a disordered atomic arrangement, which gives rise to only a first nearest neighbor peak in the simulated EXAFS spectra due to the presence of only short-range order, and at the same time does not give rise to an XRD peak. This phase undergoes an atomic rearrangement, which is characterized by a rough layer-by-layer-like atomic arrangement, in particular, the alternate stacking of Te and cation layers as well as Te termination at the vdW gaps. But the x and y positions still differ from ideal values, while deviations along the z-axis also remain. This situation is schematically shown in Fig. 4d as *cry.1*. On the other hand, this model already possesses the potential to give rise to out-of-plane XRD reflections. This can be regarded as a 3D to quasi-2D transition in chemical bonding. In short, the self-organizational tendency for atomic rearrangement derives from the inherent 2D nature of the system. Even though the *cry.1* phase has sufficient order in the z-direction to give rise to XRD reflections, it lacks the coherence derived from medium- or long-range order which leads to the absence of a second nearest neighbor peak in EXAFS. Note that an obvious candidate for the intermediate phase might be when a fraction of Cr and/or Ge atoms are still located inside the vdW gap regions. This configuration leads to similar results. Finally, all atoms settle into stable positions upon further annealing. In other words, the dominant x- or y-displacements gradually diminish, resulting in the enhancement of interference effects between the second nearest neighbor atoms and the absorbing atom. Only after the *cry.2* phase with long-range order is formed, does the second nearest neighbor peak occur in the EXAFS spectrum.

In conclusion, a dimensional change in chemical bonding during the crystallization of the amorphous phase was demonstrated using Cr_2_Ge_2_Te_6_ as a prototypical example based on two complementary structural analysis techniques as well as first principles simulations. It was found that the 3D-bonded amorphous film undergoes a phase change into a 2D-bonded crystalline system via a quasi-layered intermediate phase, which is characterized by the presence of “long-range order and short-range disorder” in atomic alignment. This concept can be generalized to other similar layered chalcogenides. In particular, since the fabrication of high-quality crystalline TMD films is required for applications, utilizing an amorphous-to-crystal transition is a potential solution. In fact, a study on the crystallization of amorphous MoTe_2_ has been recently reported^33^. Moreover, reports on 2D amorphous carbon have appeared very recently, underscoring the increased interest in disorder in the low dimension materials^34^. It should be noted that some layered materials, such as Sb_2_Te_3_, have been pointed out not to be rigorous van der Waals solids as the spacing across the vdW gaps is shorter than suggested by the vdW radius of the constituent atoms, suggesting stronger interactions^35^. Based on the DFT simulations of the interlayer binding energy^36,37^, the observation of stacking defects^38,39^, and the estimation of the Te–Te gap spacing, Cr_2_Ge_2_Te_6_ appears more likely to possess the characteristics of vdW solids. In either case, we believe that the present work will pave the way to the understanding of the crystallization mechanism of other layered materials and hence contribute to the fabrication of high-quality functional layered materials for future electronics applications.

During the review process, recently we came across a similar reference about 2D to 3D structural transformation in CrSiTe_3_. We have added this reference to the revised manuscript^40^.

## Methods

### Sample fabrication

Thin film samples of Cr_2_Ge_2_Te_6_ were prepared by RF-magnetron sputtering (MPS, ULVAC) using Cr, Ge, and Te pure metal targets. The base pressure before deposition was below 6.0 × 10^–5^ Pa, and the Ar working pressure was 0.38 Pa. The composition of the films was analyzed using an energy dispersive X-ray (EDX) spectroscopy system attached to a transmission electron microscope (JEOL, JEM2100), and was determined to be Cr_19.2_Ge_20.6_Te_60.2_ a composition with less than 1 at% deviation from the ideal composition (Cr_20.0_Ge_20.0_Te_60.0_). The as-deposited samples were divided into pieces and heated up to 150, 250, 270, 290, 320, 330, and 380 °C with a ramp rate of 10 °C/min in an Ar atmosphere, upon which the heater was switched off and the samples were allowed to cool to room temperature without being held at elevated temperature.

### XRD

X-ray diffraction (XRD) measurements were carried out at room temperature to analyze the crystal structures of the annealed samples (Rigaku, Ultima). Cu-K*α* (wavelength: 0.154061 nm) radiation was used, and *θ*/2*θ* symmetry scans (Bragg–Brentano geometry) were performed. For the XRD measurements, 200-nm-thick Cr_2_Ge_2_Te_6_ films were grown onto Si substrates covered with a thermal oxide (100 nm), followed by the deposition of a carbon layer a few nm thick immediately after removal from the sputtering chamber. The XRD patterns of the amorphous and crystalline phases were simulated using the CrystalDiffract program^41^.

### EXAFS

Extended X-ray absorption fine structure (EXAFS) measurements were carried out at the *K*-edges of Cr, Ge, and Te in transmission mode below 10 K using the cryocooler at beamline BL01B1 at SPring-8. The Si(111) and Si(311) settings of the double-crystal monochromators were used for Cr *K*-edge and Ge or Te *K*-edge, respectively. For the measurements, 1.3-μm-thick Cr_2_Ge_2_Te_6_ films were deposited onto both sides of aluminum foil. Subsequently, a Si–N protection layer of 50 nm was deposited in a different chamber immediately after breaking the vacuum. The sample was divided into five pieces with different pieces heated up to 250, 290, 330, and 380 °C under the same conditions described previously. The samples were measured in transmission stacked to a thickness sufficient to obtain an edge jump of unity at each absorption edge. The obtained EXAFS data were analyzed using the real-space multiple scattering code FEFF and IFEFFIT^42,43^. FEFF is an ab initio multiple-scattering code for calculating excitation spectra and electronic structure of the materials. This is a fully relativistic and all-electron Green function code. In the FEFF calculations, the cluster radius was set to 8 Å around the central atom.

### DFT

Density functional theory (DFT) simulations were performed to generate a melt-quenched Cr_2_Ge_2_Te_6_ model. The Vienna ab initio simulation package (VASP) was used with projector-augmented wave (PAW) pseudopotentials^44,45^. The generalized gradient approximation (GGA) of Perdew–Burke–Ernzerhof (PBE) exchange–correlation functional^46^ was utilized with a cutoff energy of 310 eV. A 120-atom supercell was created, and the Gamma point was used for Brillouin zone integration^47^. The DFT-D3 dispersion correction was used to take into account van der Waals interactions^48^. The model was melt-quenched using ab initio molecular dynamics based on a Nosé–Hoover chain thermostat. The molten phase was obtained by heating a crystalline supercell of 120 atoms up to 5000 K for 5 ps to completely disorder the system followed by cooling to 1500 K, but above the melting point over a period of 50 ps. The model was then cooled from 1500 to 300 K over a period of 5 ps and was maintained at 300 K for 10 ps to generate the disordered melt-quenched phase.

## Supplementary Information

Supplementary Figures.

## Data Availability

Data related to the figures can be found online. Other data related to this work are available from the authors upon reasonable request.
