# Supplementary material for: Dimensional transformation of chemical bonding during crystallization in a layered chalcogenide material
Source: Sci Rep. 2021 Mar 8;11:4782. doi: 10.1038/s41598-020-80301-5 (PMC7940477; doi:10.1038/s41598-020-80301-5)
Supplement: Supplementary file 1 — Supplementary Figures. [file 41598_2020_80301_MOESM1_ESM.pdf]

Supplementary information

Dimensional transformation of chemical bonding during crystallization in the layered chalcogenide material

Yuta Saito[1,5], Shogo Hatayama[2,5], Yi Shuang[2], Paul Fons[1,3], Alexander V. Kolobov[1,4], and Yuji Sutou[2]**

[1] *Device Technology Research Institute, National Institute of Advanced Industrial Science and Technology, Tsukuba Central 5, Higashi 1-1-1, Tsukuba 305-8565, Japan.*

[2] *Department of Materials Science, Graduate School of Engineering, Tohoku University, 6-6-11 Aoba-yama, Sendai 980-8579, Japan.*

[3] *Department of Electronics and Information Engineering, Faculty of Science and Technology, Keio University, 3-14-1 Hiyoshi, Kohoku-ku, Yokohama, Kanagawa 223-8522, Japan.*

[4] *Department of Physical Electronics, Faculty of Physics, Herzen State Pedagogical University of Russia, 48 Moika Embankment, St Petersburg 191186, Russia.*

[5] *These authors contributed equally.*

* Corresponding authors: yuta-saito@aist.go.jp, ysutou@material.tohoku.ac.jp

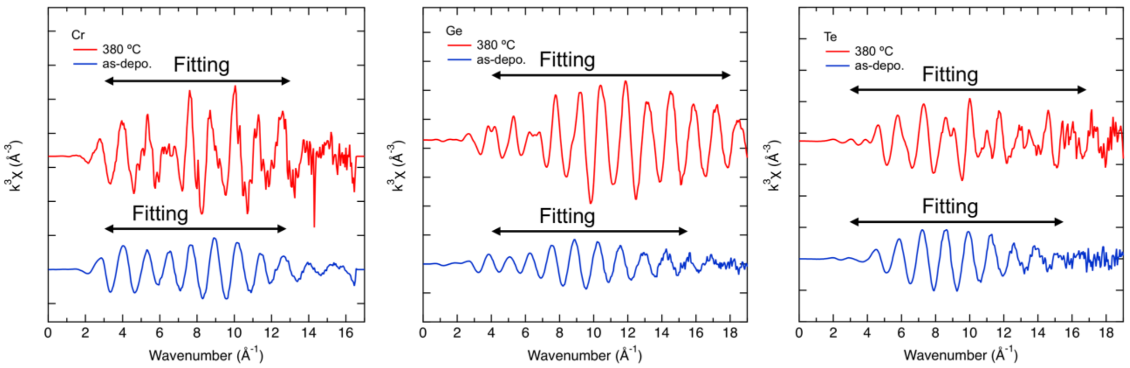

Supplementary Fig. 1 Raw data of EXAFS measurements.

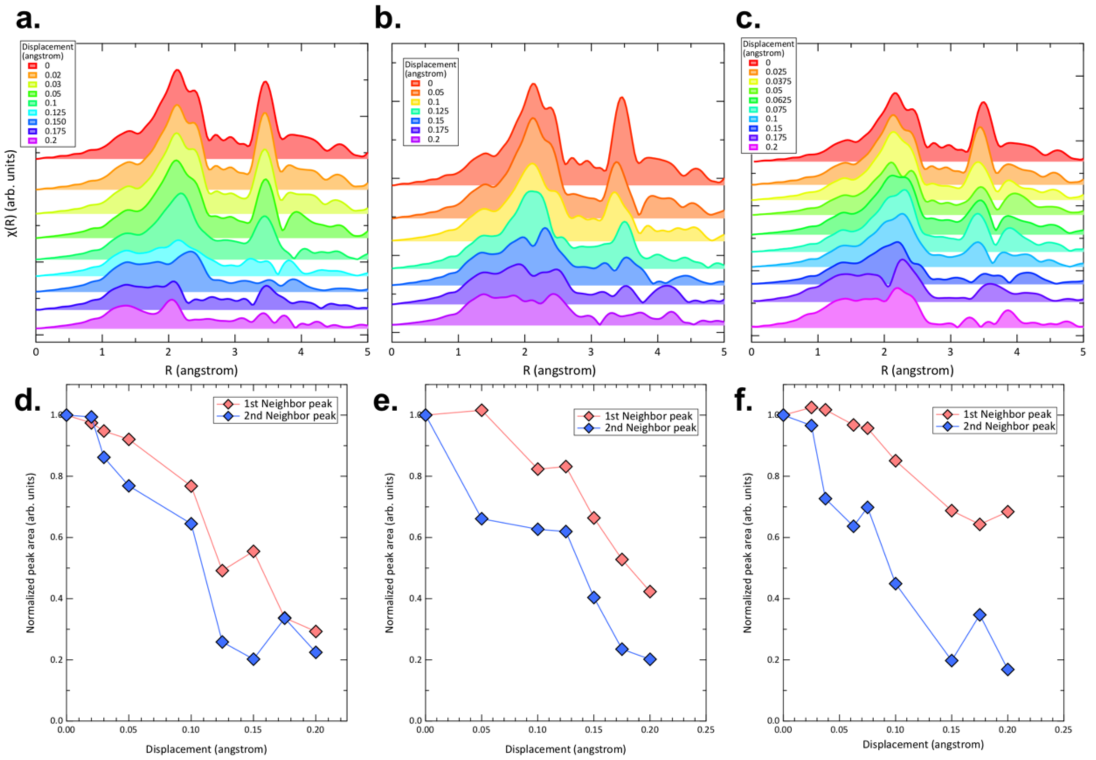

Supplementary Fig. 2 Simulated EXAFS spectra dependence with different forms of displacement. (a) All atoms displaced toward x, y, and z directions randomly. (b) Only Te atoms displaced toward x, y, and z directions randomly. (c) Only Te atoms displaced within the x-y plane randomly. (d)~(f) Corresponds normalized peak area of the first and second nearest neighbor peaks in (a)~(c), respectively.
